# Image copy-move forgery detection and localization based on super-BPD segmentation and DCNN

**DOI:** 10.1038/s41598-022-19325-y

**Published:** 2022-09-02

**Authors:** Qianwen Li, Chengyou Wang, Xiao Zhou, Zhiliang Qin

**Affiliations:** 1 School of Mechanical, Electrical and Information Engineering, Shandong University, Weihai, 264209 China; 2 Weihai Beiyang Electric Group Co. Ltd., Weihai, 264209 China

**Keywords:** Electrical and electronic engineering, Engineering, Mathematics and computing, Computer science, Information technology

## Abstract

With the increasing importance of image information, image forgery seriously threatens the security of image content. Copy-move forgery detection (CMFD) is a greater challenge because its abnormality is smaller than other forgeries. To solve the problem that the detection results of the most image CMFD based on convolutional neural networks (CNN) have relatively low accuracy, an image copy-move forgery detection and localization based on super boundary-to-pixel direction (super-BPD) segmentation and deep CNN (DCNN) is proposed: SD-Net. Firstly, the segmentation technology is used to enhance the connection between the same or similar image blocks, improving the detection accuracy. Secondly, DCNN is used to extract image features, replacing conventional hand-crafted features with automatic learning features. The feature pyramid is used to improve the robustness to the scaling attack. Thirdly, the image BPD information is used to optimize the edges of rough detected image and obtain final detected image. The experiments proved that the SD-Net could detect and locate multiple, rotated, and scaling forgery well, especially large-level scaling forgery. Compared with other methods, the SD-Net is more accurately located and robust to various post-processing operations: brightness change, contrast adjustments, color reduction, image blurring, JPEG compression, and noise adding.

## Introduction

With the image editing software becoming prevalent, such as Adobe Photoshop and ACDSee Photo Editor, people alter the content of images arbitrarily and easily. This results in the authenticity and integrity of images being questioned^1^. The question is fatal in many critical fields, especially the fields depending on image content^2^. For example, tampered images in the judiciary may affect the judgment of judges, while tampered images in news may cause political conflict^3^.

Therefore, the image forensics technique, aiming at detecting and locating the forgery, has important research value^4^. Copy-move forgery detection (CMFD) is one of the passive forensics technique for copy-move forgery (CMF). CMF is a common and easy image forgery manner, which copies and pastes a region from an image to the same image^5^. However, the tampered region in CMF is from the image itself and has the similar characteristics to the whole image, leading to the difficulty of being recognized accurately. Therefore, CMFD is a challenging topic^6^.

In the current methods, the conventional CMFD based on keypoint or block needs to build hand-crafted features and may limit one or some certain datasets. Therefore, the CMFD based on convolutional neural networks (CNN) is emerged, which could learn the features of suitable CMFD by itself. However, in the CMFD based on CNN, since the CNN loses details information easily, the accuracy of location results is lower, especially on edge.

To improve the accuracy, this paper proposes an image CMFD based on super boundary-to-pixel direction (super-BPD) segmentation and deep CNN (DCNN): SD-Net. To obtain suitable and global CMFD features, DCNN is used to extract image features, replacing conventional hand-crafted features with automatic learning. To improve the edge accuracy, a segmentation method, super-BPD, is used to extract image edge information. The proposed method SD-Net could more accurately detect and locate multiple, rotated, and scaling forgery well, especially large-level scaling forgery.

## Related works

Conventional CMFD methods mainly have two categories: block-based and keypoint-based. In block-based methods, the images are divided into many blocks, e.g. overlapping or non-overlapping, regular or irregular. The features of all blocks are extracted to represent the information, such as discrete cosine transform (DCT)^7,8^, singular value decomposition (SVD)^9^, histogram of oriented gradients (HOG)^10^, Zernike moment (ZM)^11^, local binary pattern (LBP)^8^, polar harmonic transform (PHT)^1^, etc. However, although the block-based methods can detect the tampered regions accurately, they have high computational complexities and low robustness to large-level rotation and scaling.

To reduce the computational complexity of block-based CMFD methods, the keypoint-based methods are proposed, using features of key points to replace that of blocks. The main key features are scale invariant feature transform (SIFT)^12^, speed-up robust feature (SURF)^1^, Harris^13^, accelerated-KAZE (A-KAZE)^14^, oriented FAST and rotated BRIEF (ORB)^15^, fast retina keypoint (FREAK)^16^, etc. However, most keypoints extraction methods extract few key points in the smooth regions, resulting in some forgeries in the smooth regions being ignored easily.

With the application of CNN in computer vision, CNN is used in the image forensics field^17^. The classification function of CNN judges the image to reveal if the image is tampered with. Methods^18–20^ used CNN to detect splicing, copy-move, and other forgery images by the abnormal traces of forgery, such as the inconsistent of noise and illumination direction in whole image. However, the abnormality of CMF is smaller than other forgeries, resulting in a poor effect on CMFD. Subsequently, methods which dedicated to detect CMFD appear. Methods^21–23^ used CNN to detect similarity and judge whether the image has been tampered with in a copy-move manner.

After that, researchers modify the output of the last module seeking to achieve the purpose of pixel-level CMFD. BusterNet^5^ is the first CNN framework specifically for CMF and the first CMFD method that distinguishes the source/target forgery regions, though the accuracy of the distinguish module is only 12%. Then, Chen et al.^24^ changed the parallel detection branch in BusterNet to a serialized branch, improving the accuracy of distinguishing source/ target forgery regions to 39.9%. AR-Net^17^ improved the accuracy of the located forgery region from 49.26% to 50.09%, through modifying the Simi-Det branch of the BusterNet. However, it is still unable to resist noise and blurring attacks, which impacts the accuracy of the detection results.

In addition to using VGG networks, such as BusterNet^5^, later Generative Adversarial Networks (GAN)^25^, InceptionNet^26^ and DenseNet^27^ are also used for feature extraction. It can be seen that researchers have made many attempts in the CNN-based CMFD, hoping to further improve the generalization and robustness of the algorithm.

Therefore, in pixel-level aspect, the CNN-based CMFD method has a number of potentials to be improved in terms of accuracy, robustness, special forgery region, and distinguishing the source/target. The proposed method focuses on solving the problems of accuracy and robustness.

## Proposed method: SD-Net

**Figure 1:**
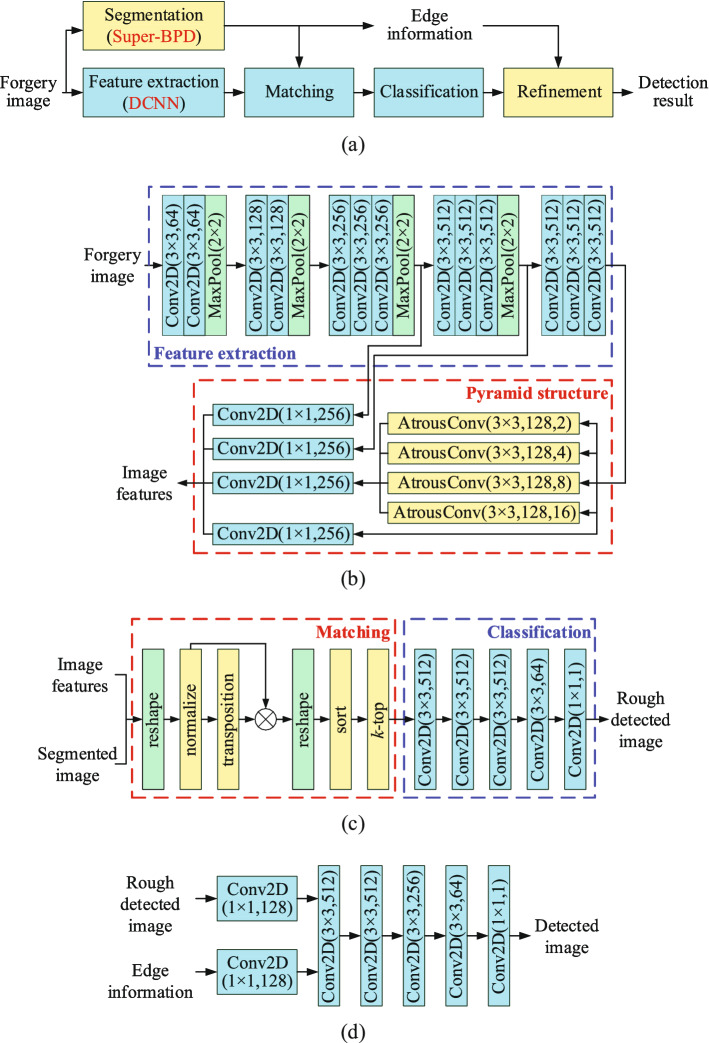
Framework of the SD-Net: (**a**) overview, (**b**) feature extraction module, (**c**) matching and classification modules, and (**d**) refinement module.

This section presents the SD-Net in detail, which flow chart is given in Fig. 1a. The SD-Net is mainly divided into five parts: segmentation, feature extraction, matching, classification, and refinement modules. Moreover, Fig. 1b–d shows the detail framework of each module of the SD-Net.

Firstly, the SD-Net uses super-BPD segmentation technology to divide a forgery image into irregular blocks, obtaining the segmented features of the image. Due to the characteristic of copy-move forgery, the pasted region is very similar to the copied region, being divided under the same or similar type of blocks. Secondly, DCNN is used to extract image features, replacing conventional hand-crafted features with automatic learning features. The feature pyramid is used to improve the robustness to the scaling attack. Thirdly, the image features are fused with the segmented features, and obtain the correlation matrix by matching module. The correlation matrix is classified and discriminated through the CNN, and the repetitive regions in the image are found out. Finally, the rough forgery detection is optimized and finetuned with BPD edge information to obtain a more refined detection result.

### Segmentation module

In the conventional image CMFD method, methods based on the combination of block and keypoints have gradually become popular^1,28^. Feature matching in the same or similar image blocks can reduce the interference of irrelevant blocks and improve the matching efficiency. On this basis, SD-Net incorporates a semantic segmentation method based on image content. After the image is segmented, feature matching is performed concerning the segmentation image. It enhances the connection between the same or similar blocks, which include both copied and pasted regions, and improves the detection accuracy.

Through the super-BPD segmentation^29^, the image is segmented by using the BPD information of the image. The BPD information $\varvec{D}_{p}$ is a two-dimensional unit vector and can be expressed as follows^29^:

1 $$\begin{aligned} \varvec{D}_ {p} = {{\overrightarrow{{B_{p}}p} } \cdot {\overrightarrow{|{B_ {p}}p|} }}, \end{aligned}$$

where $\overrightarrow{{B_{p}}p}$ is the vector pointing from the nearest boundary pixel $B_{p}$ to each pixel *p*, and $\overrightarrow{|{B_{p}}p|}$ is their distance.

Compared with other segmentation, super-BPD improves the speed while achieving high accuracy. When providing high-precision detection results, it has a lower impact on the complexity for the SD-Net.

**Figure 2:**
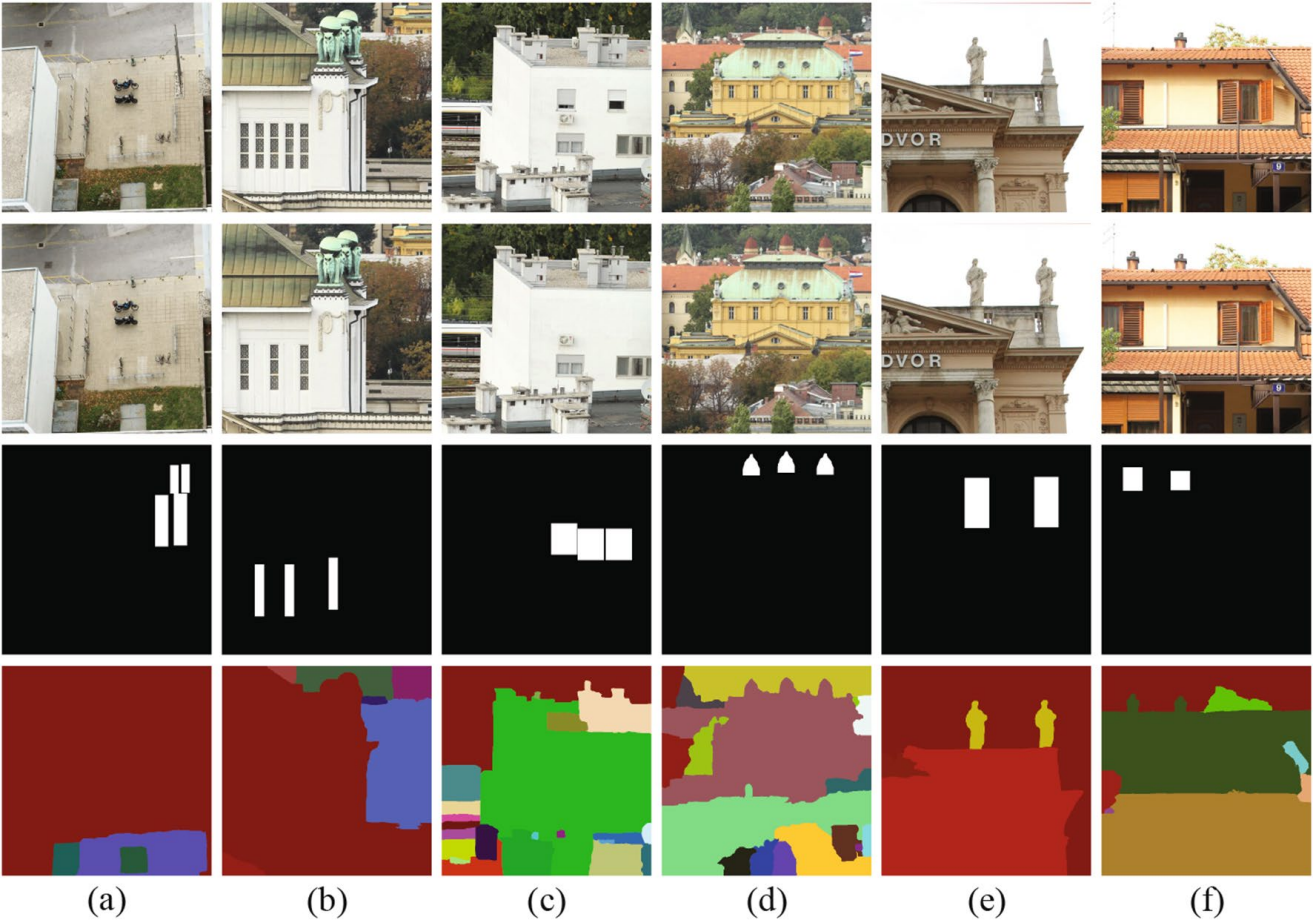
Segmentation results for super-BPD on six images in CoMoFoD^30^ datasets. 1st row: original images; 2nd row: forgery images; 3rd row: ground-truth forgery regions; 4th row: segmentation results of the super-BPD. (**a**) 002_F, (**b**) 038_F, (**c**) 030_F, (**d**) 025_F, (**e**) 012_F, and (**f**) 123_F.

Figure 2 shows six examples of the super-BPD segmentation on the CoMoFoD^30^ datasets. The 1st row is the original images, the 2nd row is forgery images, the 3rd row is ground-truth forgery regions, and the 4th row is the segmentation results of the super-BPD.

The forgeries of 002_F, 038_F, 030_F, and 025_F, shown in Fig. 2a–d, respectively, occur in regular or irregular regions with multiple pasted. The segmentation results in Fig. 2 show the segmentation module of the SD-Net will divide the copied and pasted regions into the same block. The forgeries of 012_F and 123_F, shown in Fig. 2e,f, respectively, occur in the regular region including irregular foreground and supplementary background. The segmentation results in Fig. 2e,f show the segmentation module of the SD-Net will divide the irregular foreground into the same or similar regions and divide the background into the same regions. Therefore, even in the case of irregular and multiple forgeries, the super-BPD segmentation method can still divide the copied and pasted regions into the same or similar blocks and achieve better performance.

### Feature extraction module

Conventional algorithms are more dedicated to hand-crafted features that are similar to the copied and pasted regions. At the same time, it also takes into account attacks such as rotation, scaling, and noise, and it is difficult to find an optimal feature descriptor. The emerging CNN methods can better solve the problem by using big data to learn features suitable for image CMFD, and avoid the limitations of hand-crafted features as much as possible.

The SD-Net uses a DCNN to extract image features, and uses VGG16^31^ as the backbone network. Figure 1b shows the specific network framework of the feature extraction module.

The blue box in Fig. 1b, which denotes feature extraction, is that the VGG16 network removes the fully connected layer to extract image features. The red box in Fig. 1b, which represents a pyramid structure, consists of the CNN shallow information and atrous spatial pyramid pooling (ASPP) layer^32^.

**Figure 3:**
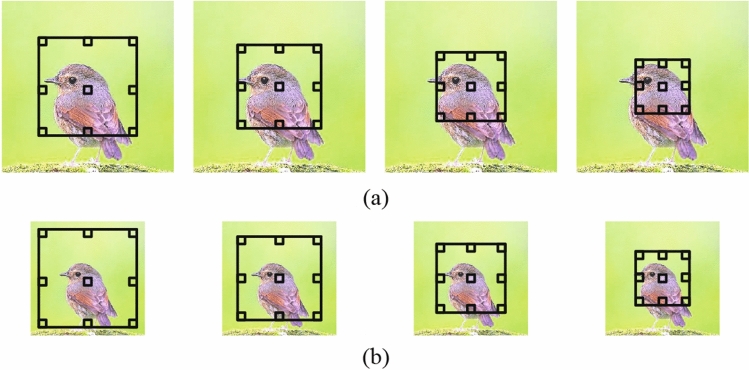
Feature in atrous spatial pyramid pooling (ASPP) on the image in CASIA II^33^ dataset: (**a**) original image and (**b**) image is scaled by 0.66.

ASPP is used to extract the multi-scale features of the image and robust to scaling^17^ by considering different object ratios. Figure 3 shows the feature in ASPP, on the image in CASIA II^33^ dataset, and the black box is the field in four $3 \times 3$ atrous convolution. Figure 3a is the original image and field in atrous convolution, while Fig. 3b is the image scaled by 0.66 and field in atrous convolution. In Fig. 3, the 1st field in Fig. 3a is similar to the 3rd in Fig. 3b. That means that there is similar feature in ASPP even though the image is large-level scaled, to detect the copy-move forgery. Therefore, the module improves detection accuracy and is capable of detecting large-level scaling forgery which conventional methods failed.

On the other hand, though the deep network increases the receptive field, it loses some local detailed information when extracting the global information of the image. In BusterNet^5^, only the final output in the whole VGG network is used without considering the local information, which cannot meet the edge accuracy requirements in the forgery detection^17^. The SD-Net takes advantage of the regularity of VGG16 to consider the local information features in the shallow network outputs, and combines it with the ASPP layer, forming the feature pyramid structure.

### Matching and classification module

The auto-correlation matching module (the red box) and the classification discriminant module (the blue box) is shown in Fig. 1c. The essence of the matching stage in CMFD is judging the similarity of two feature vectors. The SD-Net uses the correlation matrix to measure the relationship between sample vectors.

The image features extracted from feature extraction module are merged with the segmentation image delivered by the segmentation module to obtain a feature matrix $\varvec{M}_{\text {f}}$. The size of $\varvec{M}_{\text {f}}$ is $[m \times n, f]$, where the $m \times n$ is the image resolution and the *f* is the dimension of pixel feature.

The correlation matrix $\varvec{M}_{{\text {cor}}}$ is obtained by follows:

2 $$\begin{aligned} {\varvec{M}_{{\mathrm {cor}}}} = {\varvec{M}_{\mathrm {f}}} \cdot \varvec{M}_{\mathrm {f}}^{\mathrm {T}}, \end{aligned}$$

where $[\cdot ]^{\text {T}}$ is the transposition operation. The size of $\varvec{M}_{{\text {cor}}}$ is $[m \times n, m \times n]$, which representing the similarity between all features. The closer the similarity is to 1, the higher the similarity between the two features, and the greater the possibility of forgery in the region as described by the feature.

Furthermore, the dimension of the correlation matrix $\varvec{M}_{{\text {cor}}}$ is changed to $[m, n, m \times n]$, and then sort the third dimension in a descending order, intercepting the second to *k*-th feature after sorting features. The reason for discarding the first similarity feature is that the maximum similarity is between the feature and itself, and approaches infinitely close to 1, which is meaningless for finding the forgery region. Moreover, it will interfere with the subsequent judgment of the matching regions.

After obtaining the correlation matrix, the SD-Net judges whether there is a similar feature vector in the region rather than looking for a matching position. Cancellation of the mapping search process reduces the complexity of the SD-Net and has advantages in the case of multiple copy-move forgeries.

The blue box in Fig. 1c is the framework of the classification discrimination module. Based on the classification function of the convolutional network, the obtained matching results, which are represented by image pixels, are distinguished whether it belongs to a forgery region.

### Refinement module

Due to the loss of detailed local information after deep convolution, the detected forgery region suffers from the loss of fine edges. Therefore, the SD-Net refines edge details, through fusing the edge information extracted from the super-BPD method and the rough detection image from the matching and classification module. The refinement network is shown in Fig. 1d.

The edge information, that is, the BPD information, is generated in the segmentation module. In the refinement module, rough detected result is combined with the edge information, increase the weight of the edge in the detection result, and get the final detection result.

Firstly, extend the rough detection image and the edge information from 2-dimension to 128-dimension, obtaining deeper feature information. Then, four convolutional layers are used to learn the detection image edges. Through the BPD edge information, add or subtract the edge in rough detection image. Finally, the $1 \times 1$ convolutional layer is used to reduce the feature dimension and obtain the detection image.

### Training details

The training strategy of the SD-Net is mainly divided into the following two steps:

1. Use the PascalContext^34^ datasets to train the image segmentation module, to obtain a better segmentation effect^29^. Then freeze the trained segmentation module parameters to ensure that they do not participate in the second step of training.
2. Use the USCISI^5^ train set (include 80,000 images) to train the image tampering detection branch, including feature extraction, auto-correlation matching, classification, and refinement modules to accurately classify the pixels in the forgery image into tampering or non-tampering classes.

Because image forgery detection is a binary classification problem, the binary cross entropy loss (BCELoss) $L_{{\text {BCE}}}$ is used for the training loss function, which is expressed as follows^17^:

3 $$\begin{aligned} {L_{{\text {BCE}}}} = - \sum \limits _{p \in \Omega } {[{y_p} \cdot \log ({{\hat{y}}_p}) + (1 - {y_p}) \cdot \log (1 - {{\hat{y}}_p})]} \end{aligned}$$

where $\Omega$ is the image domain, $y_p \in \{0,1\}$ represents the ground-truth for the pixel, while ${{\hat{y}}_p}$ represents the predicted result of the SD-Net for the pixel.

## Experimental results and discussions

This section first introduces the datasets and evaluation metrics used in all experiments. Following that, a series of validation experiments are conducted to evaluate and discuss the performance of the SD-Net: ablation experiments, robustness experiments, and compare the SD-Net with the state-of-the-art methods. Finally, complexity of the SD-Net is analysed.

The SD-Net is compared with the six state-of-the-art methods: conventional block-based^35^, conventional keypoint-based^36^, combined keypoint and block^1^, and CNN-based^5,17,37^ CMFD methods. Wu et al.^37^ detects forgery according to trace of manipulation, while BusterNet^5^ and AR-Net^17^ detect forgery according to similarity regions. In BusterNet, the Simi-Det branch uses VGG16 to extract features, which is the basic framework in feature extraction of the SD-Net. In AR-Net, the ASPP module is used to extract multi-scale features, similar to the SD-Net.

All experiments in this paper are performed on a 64-bit win10 PC with the Intel Core i9-9960X CPU @ 3.10GHz, 64GB RAM, and two parallel NVIDIA GeForce RTX 2080 Ti GPUs.

### Datasets and evaluation metrics

To test generalization, USCISI test set (include 20,000 images)^5^, CoMoFoD (include 5000 images)^30^, and the copy-move forgery images in CASIA II (include 1313 images)^33^, a total of 26,313 images, are used for testing the SD-Net.

In CMFD methods, the precision *p*, recall *r*, and *F* score metrics are commonly used to evaluate the performance of methods and are defined as follows^1^:

4 $$\begin{aligned} p = \frac{N_{\text {TP}}}{N_{\text {TP}} + N_{\text {FP}}},{\text { }} r = \frac{N_{\text {TP}}}{N_{\text {TP}} + N_{\text {FN}}},{\text { }} F = 2 \cdot \frac{{p \cdot r}}{{p + r}}, \end{aligned}$$

where $N_{\text {TP}}$ is the number of pixels that predict tampered pixels as tampered pixels; $N_{\text {FP}}$ is the number of pixels that predict original pixels as tampered pixels; $N_{\text {FN}}$ is the number of pixels that predict tampered pixels as original pixels.

The three metrics are used to evaluate the performance of the SD-Net and other methods. If the precision *p*, recall *r*, and *F* are larger, it means that the image CMFD algorithm locates the repeated regions more accurately. If the precision *p* is low, it means that the detected tampered region is smaller than correct; if the recall *r* is low, it means that the detected tampered region is larger than correct; the *F* score comprehensively considers the precision and recall, which can fully reflect the performance of the detection methods.

### Validation of the SD-net

To validate the SD-Net, the ablation experiments and robustness experiments are conducted to compare the SD-Net with the state-of-the-art methods, and then analyse complexity of the SD-Net.

#### Ablation experiment

To prove the effectiveness of the component frameworks in the SD-Net, such as segmentation and optimization, the ablation experiments were carried out for each component.

In ablation experiments, the SD-Net are tested on the USCISI^5^ test set. Table 1 shows the detection results of the ablation experiments on the USCISI^5^ test set. Moreover, in Table 1, “Base-Refine” means the framework with only the refinement module, “Base-Segment” means the framework with only the segmentation module, and “Base-Segment-Refine” means the framework with the segmentation and refinement modules, which is the SD-Net.

**Table 1:** Results of the ablation experiments for the SD-Net.

Methods	*p*	*r*	*F*
Base-refine	0.78	0.92	0.82
Base-segment	0.75	0.85	0.78
Base-segment-refine	0.91	0.88	0.89

**Figure 4:**
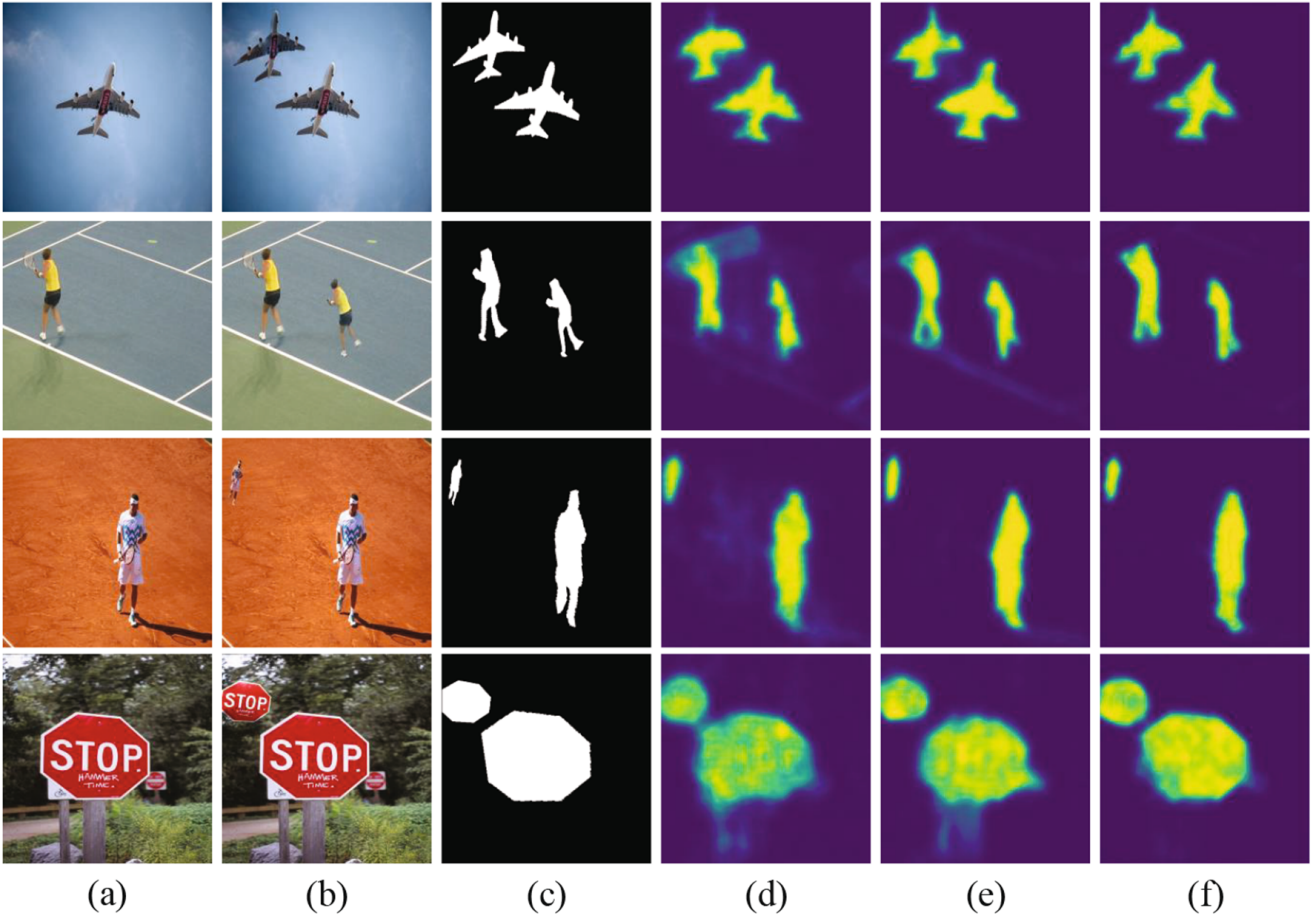
Detection results of the SD-Net on six copy-move forgery images in USCISI^5^ datasets: (**a**) original images, (**b**) forgery images, (**c**) ground-truth tampered regions, (**d**) detection results of Base-Refine, (**e**) detection results of Base-Segment, and (**f**) detection results of the SD-Net.

From Table 1, the *p* of Base-Segment-Refine is higher 0.13 and 0.16 than that of Base-Refine and Base-Segment, respectively. the *F* of Base-Segment-Refine is higher 0.07 and 0.11 than that of Base-Refine and Base-Segment, respectively. It means that the refinement and segmentation modules improve the detected results, especially the precision *p*. The *r* of Base-Segment-Refine is lower 0.04 than that of Base-Refine. The reason is that the segmentation module enhances the connection between the same blocks, and may bring some false matching whose spatial distance is too short. For the purpose of clarity, detection results of the SD-Net on six copy-move forgery images in USCISI^5^ are shown in Fig. 4.

It can be seen from the difference between Fig. 4d,f that the segmentation module can improve the detection accuracy and reduce ghosting. It can be seen from the difference between Fig. 4e,f that the refinement module can refine edge.

In Fig. 4, the tampered regions are occurred rotation-only (the 1st row), scaling-only (the 2nd row), rotation and large-level scaling (the 3rd row), and large-level scaling-only (the 4th row). Figure 4 shows the SD-Net can handle rotation and scaling well, especially large-level scaling, owing to the multi-scale features extracted by the ASPP module. However, the 3rd row in Fig. 4 shows that the SD-Net detects the small tampered regions, which, however, do not have sufficiently refined edges, an effect which needs to be improved in the future.

#### Robustness experiment

To test the robustness of the SD-Net, the experiment is conducted on CoMoFoD^30^ datasets, which include forgery images with six post-processing operations: brightness change, contrast adjustments, color reduction, image blurring, JPEG compression, and noise adding. Details of the six post-processing operations can be found in CoMoFoD^30^.

**Figure 5:**
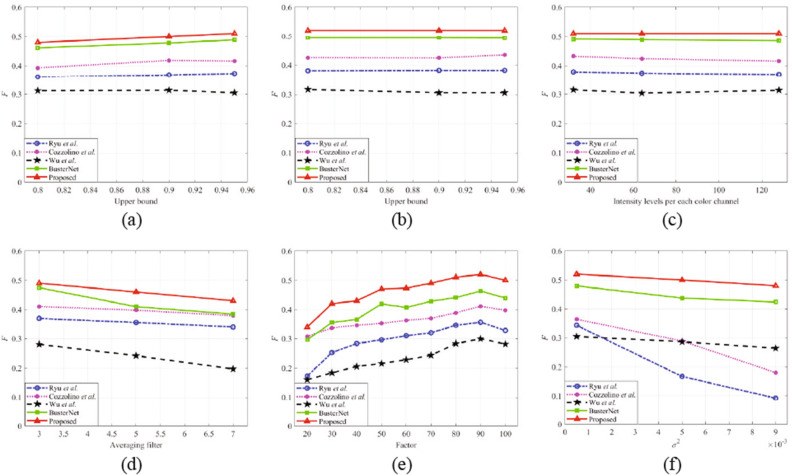
The *F* average of the SD-Net and other CMFD methods under six post-processing: (**a**) brightness change, (**b**) contrast adjustments, (**c**) color reduction, (**d**) image blurring, (**e**) JPEG compression, and (**f**) noise adding.

In robustness experiments, the SD-Net are trained on USCISI^5^ train set and tested on CoMoFoD^30^ datasets. Figure 5 shows the *F* average of the SD-Net and other CMFD methods under six post-processing operations in CoMoFoD^30^. Meanwhile, the robustness of the SD-Net is compared with the four state-of-the-art methods.

From Fig. 5, the robustness of the SD-Net is better than that of other methods, especially the robustness to image blurring, JPEG compression, and noise adding post-processing operations. The *F* of detection results of the SD-Net is similar to that of BusterNet^5^, due to the similar CNN basic framework in feature extraction. The *F* of detection results of the SD-Net is better than that of the conventional hand-crafted features^35,36^, because these hand-crafted features are affected by attacks relatively large. The *F* of detection results of Wu et al.^37^ is the worst since the trace of manipulation is affected by post-processing operation easily.

#### Comparison with the state-of-the-art methods

To evaluate and discuss the performance of the SD-Net, the comparison experiments are conducted on CoMoFoD^30^ and CASIA II^33^ datasets, which is also used in BusterNet^5^ and AR-Net^17^.

In robustness experiments, the SD-Net are trained on USCISI^5^ train set and tested on CoMoFoD^30^ and CASIA II^33^ datasets. Table 2 shows the detection results comparison in terms of average *p*, *r*, and *F* between the SD-Net and other six methods on CoMoFoD^30^ and CASIA II^33^ datasets. The *p*, *r*, and *F* of the compared methods are derived from AR-Net^17^ and the bold values denote the greatest performance in the six methods.

**Table 2:** Detection results compison in terms of average *p*, *r*, and *F* (%) between the SD-Net and other methods on CoMoFoD^30^ and CASIA II^33^ datasets.

Methods		CoMoFoD^30^			CASIA II^33^		
		*p*	*r*	*F*	*p*	*r*	*F*
Conventional	Ryu et al.^35^	45.78	34.35	37.37	22.71	13.36	16.40
	Cozzolino et al.^36^	39.92	47.61	41.83	24.92	26.81	25.43
	Wang et al.^1^	49.09	57.45	46.44	30.64	31.23	31.08
CNN-based	Wu et al.^37^	36.29	40.41	31.13	23.97	13.79	14.64
	BusterNet^5^	57.34	49.39	49.26	55.71	43.83	45.56
	AR-Net^17^	54.21	46.55	50.09	**58.32**	37.33	45.52
	SD-Net	**59.11**	**57.69**	**50.77**	57.48	**51.25**	**48.06**

Maximum values are in bold.

From Table 2, the SD-Net achieves better performance as compared with conventional methods^1,35,36^, since the hand-crafted features in conventional methods are more suitable for a specific datasets which they are designed for. The SD-Net performs significantly better than Wu *et al*.^37^, due to the trace of manipulation is what copy-move forgery is difficult to detect. The SD-Net shows a remarkable gain over BusterNet^5^ and AR-Net^17^, due to the segmentation and edge refinement modules. However, the *p* of detection results of AR-Net^17^ on CASIA II^33^ datasets is higher than that of the SD-Net, bacause the AR-Net detection results are smaller than ground-truth tampered regions.

**Figure 6:**
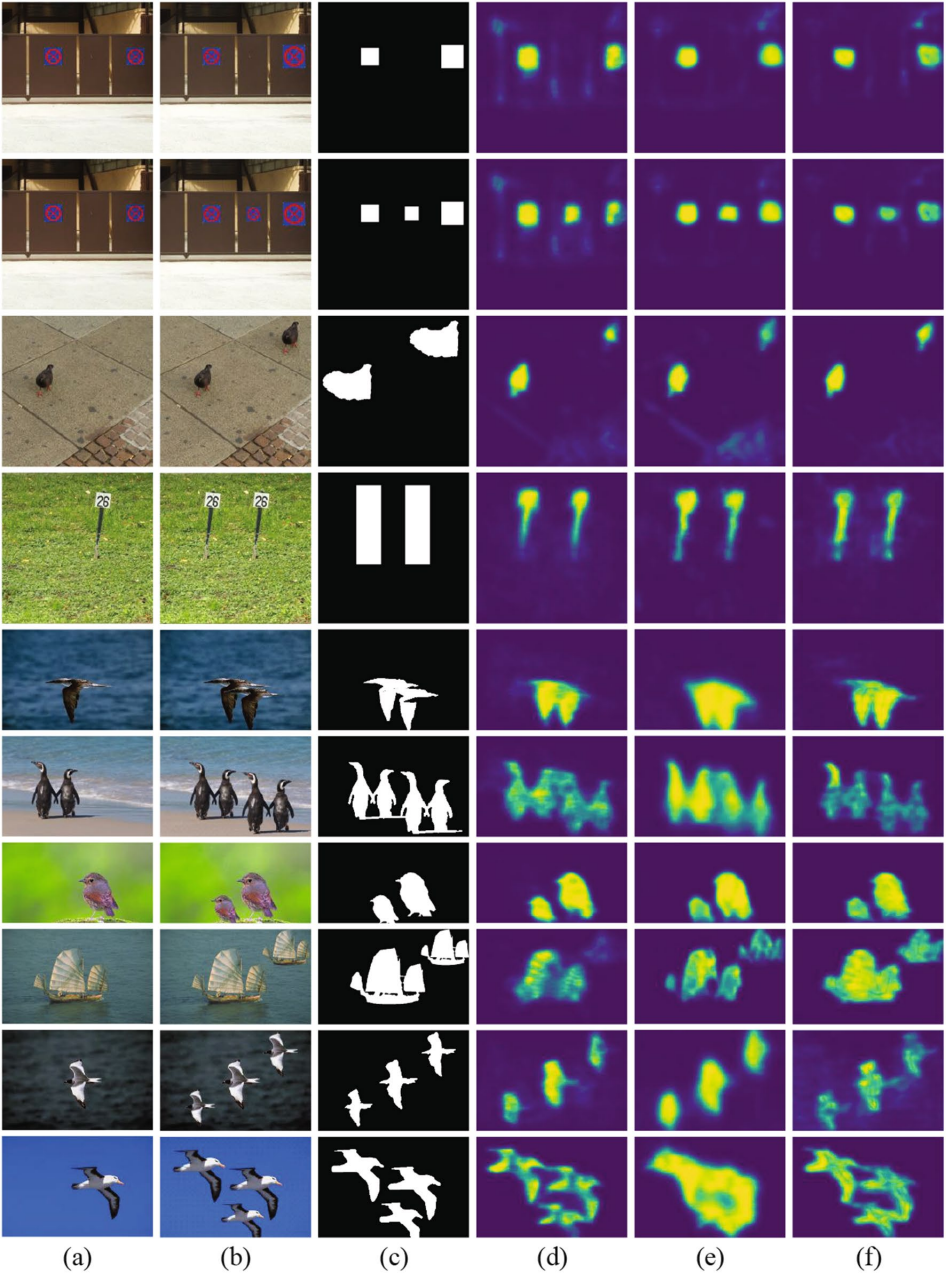
Detection results of the SD-Net on CoMoFoD^30^ and CASIA II^33^ datasets: (**a**) original images, (**b**) forgery images, (**c**) ground-truth tampered regions, (**d**) detection results of Base-Refine, (**e**) detection results of Base-Segment, and (**f**) detection results of the SD-Net.

To observe the subjective effect, the detection results of the SD-Net on ten copy-move forgery images in CoMoFoD^30^ and CASIA II^33^ datasets are shown in Fig. 6. The 1st to 4th rows images are from CoMoFoD^30^ datasets and the 5th to 10th rows images are from CASIA II^33^ datasets.

The 1st and 2nd rows in Fig. 6 show that the forgery occurring in single and multiple regions could be detected well. However, the 3rd and 4th rows in Fig. 6 show that the SD-Net detects only the object without background when the forgery occurred in obvious objects with a part of the background.

The 5th and 6th rows in Fig. 6 show the SD-Net detects forgery well, except the forgery occurred in very narrow edges. The reason is that the deep convolution network will discard some details and the segmentation module will weak the matching in block edges. The 7th and 8th rows in Fig. 6 show the large-level scaling forgery could be detected well, due to the ASPP module. The 9th and 10th rows in Fig. 6 show that the forgery in multiple regions could be detected, but the detection results have some shadows from similar backgrounds and could ignore narrow edges.

Compared with other methods, such as BusterNet^5^ and AR-Net^17^, the detection images of SD-Net are more accurate, but there are background shadow, which need to be improved in the future.

#### Complexity analysis

To measure the effectiveness of the SD-Net, complexity analysis is conducted, including time complexity and space complexity. Because the training strategy of the SD-Net is divided into two steps, the complexity analysis is obtained by adding the two steps.

The time complexity is represented by the number of floating-point operations (FLOPs) and calculated as follows:

5 $$\begin{aligned} \mathrm{{Time}} \sim O\left( {\sum \limits _{l = 1}^d {M_l^2 \cdot K_l^2 \cdot {C_{l - 1}} \cdot {C_l}} } \right) , \end{aligned}$$

where *d* is the number of convolutional layers, $M_l$, $K_l$, and $C_l$ are the output feature map size, kernel size, and number of channels of the *l*-th layer convolution, respectively. The number of FLOPs of the SD-Net can be divided into the sum of the Step (1) and Step (2). When the input image is $512 \times 512 \times 3$, the time complexity of the SD-Net is shown in Table 3.

Space complexity, that is, the size of the memory consumption, including the training parameters and the output feature map size of each layer, and could be calculated as follows:

6 $$\begin{aligned} \mathrm{{Space}} \sim O\left( {\sum \limits _{l = 1}^d {K_l^2 \cdot {C_{l - 1}} \cdot {C_l}} + \sum \limits _{l = 1}^d {M_l^2 \cdot {C_l}} } \right) , \end{aligned}$$

The memory consumption of the SD-Net can be divided into the sum of the Step (1) and Step (2). When the input image is $512 \times 512 \times 3$, the space complexity of the SD-Net is shown in Table 3.

**Table 3:** The complexity comparison between the SD-Net and BusterNet^5^.

Complexity	SD-Net			BusterNet^5^
	Step (1)	Step (2)	Total	
Number of operations (G)	1450	97.47	1547.47	146.66
Amount of training parameters (M)	28.01	18.32	46.33	15.30
Memory consumption (MB)	4320.41	827.48	5147.89	2515.92

In Table 3, the complexity of the SD-Net is compared with BusterNet^5^. The Step (2) of the SD-Net does not divide the source/target regions for tamper detection, so the time and space complexity of the Step (2) are lower than those of BusterNet^5^. However, since the SD-Net contains a Super-BPD segmentation module (Step (1)), which re-extracts edge information in the tampered image, which greatly increases the number of operations and memory consumption, the complexity of the SD-Net is higher than that of BusterNet^5^.

## Conclusions

SD-Net is proposed to solve the problem that the detection results of the most CNN-based CMFD methods have relatively low accuracy. The super-BPD segmentation technology is used to improve edge detection accuracy. The DCNN is used to improve method robustness. The experiments show that SD-Net is more accurately located in edge and robust, especially large-level scaling forgery. However, the SD-Net introduced the segmentation module and dual-branch structure, resulting in the method being more complex. The method that reduce complexity while ensuring accuracy is need be investigated in the future. Moreover, detecting forgery with similar but real regions also requires deep exploration.

## Data Availability

The datasets generated and/or analysed during the current study are available in the GitHub repository, [https://github.com/lalalalqw/SD-Net]. The datasets used and/or analysed during the current study available from the corresponding author on reasonable request.
